# Framing the mobility transition: public communication of industry, science, media, and politics in Germany

**DOI:** 10.1186/s13705-022-00374-0

**Published:** 2022-12-27

**Authors:** C. E. Drexler, B. Verse, A. Hauslbauer, J. Lopez, S. Haider

**Affiliations:** Boysen-TU Dresden-Research Training Group, 01062 Dresden, Germany

**Keywords:** Multi-Level Perspective (MLP), Framing, Verkehrswende, Mobilitätswende, Transport, Mobility, Germany

## Abstract

**Background:**

Applying the Multi-Level Perspective (MLP) on socio-technical transitions, paired with the interdisciplinary framing approach, this paper investigates how incumbent actors of automobility in Germany framed the issue of a "transition of mobility and transport" ("Verkehrs/Mobilitätswende") in their public communication in 2020. We first identified representatives of industry, science, policy, and media, since the Verkehrs/Mobilitätswende and its implementation measures are contested among these actors. Employing qualitative content analysis, we then screened 325 public documents according to the elements of the framing approach *problem definition, causal interpretation*, *moral evaluation*, and *treatment recommendation*.

**Results:**

Findings show that most of the actors frame a transformation of transport and mobility as a necessity. Their arguments encompass environmental and climate-related issues as well as infrastructural problems for bikes and public transport caused by the hegemony of automobility. The actors propose a variety of solutions, primarily focusing on technical innovations for cars or on the expansion of different infrastructures to achieve a modal shift towards sustainable mobility.

**Conclusion:**

This paper demonstrates that there is no common understanding of the problems and solutions to foster a mobility transition, as the diversity of problems and solutions proposed within the frame elements is high and complicates the prevailing implementation gap of the mobility transition. Therefore, MLP should be conceptually and methodologically bridged with the interdisciplinary framing approach, particularly with regard to the transition of mobility and transport.

## Background

As in many other countries, automobility has developed as the dominant mobility culture in Germany [1]. Private and fossil fuel-powered cars are still the most widely used mode of transport, whereas the share of bicycles, pedestrians, and public transport accounts for less than one-quarter of the total modal share [2, 3]. These developments lead to socio-political challenges on a global and local scale. About 23 percent of worldwide greenhouse gas emissions are attributed to the transport sector [4]. In Germany, the transport sector is the third-largest source of CO_2_ emissions [5]. Simultaneously, this is the only sector that has not reduced its emissions in recent years [6]. Additionally, the consequences of traffic accidents, traffic noise, other air pollutants, and traffic jams represent a burden on human health and the environment, especially in urban areas [7]. Furthermore, the developments of technical and social innovations indicate a need to rethink and redesign traffic and mobility systems towards sustainability.

This is also reflected in Germany's public and political discourse in the run-up to the federal elections in the fall of 2021—many actors from science, industry, media and politics were tabling their stakes and arguing about solutions and measures to foster a sustainable transport system in Germany. The notion of a transport and mobility transition, known in German as Verkehrs/Mobilitätswende has increasingly appeared on the public and political agenda. As the global Covid-19 pandemic was also affecting the mobility of German citizens, the deficits and challenges to spur more environmentally and socially inclusive mobility became apparent.

Since Germany's prevailing mass media and political discourse focused on the hegemony of automobility, attention was only occasionally drawn to the necessity of transforming transport and mobility or questioning its existence at all. The future was portrayed as perpetuating the current state, coloured by technical innovations [8–10]. In order to investigate socio-technical transition processes in the transport sector, as accumulated in the notion of a mobility transition, it is therefore essential to analyse how different actors in the dominant regime as well as in the various niches communicate about the mobility transition.

In Germany, although 81 per cent of the German population ages 18 to 67 favour a Verkehrswende towards sustainable mobility [11], it is not always clear what measures are necessary to achieve the transition this support refers to. The term "Verkehrswende" or lately "Mobilitätswende" came up to address and capture the transition of (1) the transport mode (*Verkehr*) and (2) mobility as a need (*Mobilität*) [12]. Both concepts can be understood as "socio-technical transformation processes in the transport sector to greatly reduce the environmental and health impacts caused by traffic to enable the sustainable development of this sector" [13] (p.110).^1^ In public discourse, however, the terms are not used distinctly. Instead, the notion of "Antriebswende" [14], which only refers to a transition of the powertrain towards e-mobility and hydrogen, rarely appears and merely captures a part of this encompassing transition. In this study, we focus on the notion of Verkehrs/Mobilitätswende. Regarding the distinction in the definition, we use the term "mobility transition" or its synonyms for the sake of readability.

### Research gap and question

Given that a multi-perspective state of knowledge on the future design of mobility and traffic already exists, discursive negotiation processes in public arenas are central for the configuration of structures, technologies, and behaviours within a mobility transition and, therefore, should be given more attention in scientific research. With a topic as complex as the mobility transition, discursive struggles defining problems and solutions are likely, as different actors have different views and interpretations of a mobility transition and how it should be conducted [11]. Due to political and economic power maintenance, mainly established actors with dominant positions in this sector are trying to shape the public discourse [15, 16]. In addition, the role of science is of particular relevance for disseminating knowledge and public discussion of research findings within transitioning processes [17]. As a platform for controversial debate and to shape public opinion by informing about news, backgrounds, contexts, and developments, media are also an essential part of the public discourse within such processes [18]. Since studies so far have barely addressed the views of different actors in the public discourse on the transition of mobility and transport, our study attempts to fill this research gap by asking:

*RQ: How do established industry actors, science, politics, and the media publicly frame the problems and solutions of the "Verkehrs/Mobilitätswende" in Germany?*

### Framing in the socio-technical transition of transport and mobility

The transition of transport and mobility is understood as a socio-technical transition, as a sequence of processes that lead to a fundamental change within the technological, material, organizational, political, socio-cultural, and economic dimensions of a system [19]. It is shaped by co-evolutionary interactions between quite different elements like infrastructures, technologies, policy regulations, a broader institutional environment, and the diverse interests, preferences, and attitudes of various groups of actors and users [20].

The analysis of *emphasis frames* [21], by which different actors communicate their views and preferences, enables an understanding of the complexity and dynamics of socio-technical transformation processes [22–25]. Frames are considered successful if attention is drawn to the actor's actions in the respective public arena and if their position, proposed solutions, and interpretations are reflected in the media reporting [26]. The successful actor can then establish the dominant frame. The framing process takes several steps: individual aspects of a topic are placed in the foreground, while others are purposefully not communicated and, therefore, remain hidden. These individual aspects are then related to each other, shaping a context to create meaningful content. Finally, connections to other topics are stated, and follow-up communication can be established accordingly [27].

Frames are dynamic and can change over time [28]. A frame defines what a perceived problem consists of, identifies which factors have caused this problem, and suggests possible solutions; it can contain the following individual components [27]:

1. *Problem definition: Determines which challenges and problems arise due to events or actions of actors. The actors involved are assumed to weigh up costs and benefits according to common cultural values and norms.*
2. *Causal interpretation*: Refers to the causes behind the respective problem or challenge.
3. *Moral evaluation:* Assessment of causal factors and their corresponding effects.
4. *Treatment recommendation*: Recommendations for action that suggest measures to solve the problem.

Since the framing approach only allows us to research statuary statements, we incorporate the Multi-Level Perspective (MLP) on socio-technical transitions [29] in our analysis. This enables us to address the research gap by considering and situating these statements within the dynamics and complexity of the transformation processes of the transport system, which are systemically depicted within the MLP. Through the combination of those two approaches, more profound conclusions can be drawn about whether and how different advocates construct mobility in transition in their public communication.

### Multi-level perspective on socio-technical transitions

In the MLP framework, transitions are viewed as nonlinear processes resulting from developments at three interrelated analytical levels: (1) the socio-technical regime includes various established social areas with corresponding actors and rules that reproduce, empower, and limit the developed system. The regime is understood as a configuration of market and consumption preferences, industry, science, culture, politics, and technologies [29]. In Germany, individual motorized transport powered by fossil fuels (i.e. the automobility regime) is the dominant regime following factors such as market share and user behaviour [3]. Other regimes are public transport or pedestrian and bicycle transport. Regimes shape and, simultaneously, are shaped by overarching, exogenous factors of the (2) socio-technical landscape, e.g. the Covid-19 pandemic [30], climate change, or the limitation of fossil resources [31]. Changes within the landscape usually take place slowly; the influence of the landscape is not seen as a deterministic influence but can favour or hinder changes within regimes and niches [32]. (3) At the niche level, socio-technical innovations, central to the design of transformation processes, emerge. Examples of socio-technical niche innovations are sharing offers (e.g. car-sharing), demand-driven offers (pooling), and Mobility as a Service (MaaS) offers.

Transformation processes arise only through the interaction of the three levels, with the regime as the centre of power and enabler of such processes [33]. If instability occurs within the regime level, for example, when actors' expectations and values differ, existing path dependencies can be broken up, creating diffusion potential for niche innovations [34]. Established actors can oppose niche innovations, hinder their development and implementation, or even prevent them entirely.

Although the MLP has been critically discussed [35], it appears to be illustrative to trace how different advocates communicate publicly (reasons for) the problems and possible solutions of Verkehrs/Mobilitätswende in Germany. This sheds insights into what is intended by the term and the potential developments from the regime. Due to the lack of extant research on our subject, we chose an explorative approach by utilizing qualitative content analysis.

## Methods

The qualitative research design we chose seems appropriate because of the exploratory nature of this study. It allows us to capture problems and solutions of the multiple interrelationships in complex systems [36]. According to the definition of a socio-technical regime, industry, science, politics, and culture, i.e. media, are established actors of automobility in Germany [30, 32]. We identified exemplary representatives as follows.

### Industry

Having the highest market share and based on their importance to the automotive regime [20, 37], the two German car manufacturers, *VW* and *BMW,* were chosen as representatives for the industry. In addition, representing automotive suppliers *Bosch* and *Continental* were selected as companies with the highest sales in the German automotive industry [38].

### Science

As they list the most scientific journals and papers, the interdisciplinary bibliographic databases *Scopus* and *ScienceDirect* were included in our study. In addition, the online database *FIDmove* was selected for the analysis, as it focuses on interdisciplinary, scientific literature covering traffic and mobility research.

### Politics

Focusing on the identification of political actors involved in policymaking [39], we included parties holding seats in the *German Bundestag* legitimized by democratic elections: *Alternative für Deutschland* (AfD), *Freie Demokratische Partei* (FDP), *Christlich Demokratische Union Deutschlands/Christlich-Soziale Union in Bayern e.V.* (CDU/CSU), *Bündnis 90/Die Grünen*, *Sozialdemokratische Partei Deutschlands* (SPD), and *Die Linke*. In addition, due to their direct responsibility for the areas of transport and mobility, the *Committee on Transport and Digital Infrastructure* (AVI) in the *German Bundestag* and the *Federal Ministry of Transport and Digital Infrastructure* (BMVI) were included within the study.

### Media

Since public opinion is shaped mainly by journalistic content [40], which also includes social categories resulting from cultural values and norms [41], we integrated all national daily newspapers in Germany as representatives of culture: *die tageszeitung* (taz), *Süddeutsche Zeitung* (SZ), *Frankfurter Allgemeine Zeitung* (FAZ), and *Die Welt*.

The official websites of each of the representatives mentioned above were selected as research subjects (see Table 1). For newspapers, we limited the material by only including opinion pieces since they reflect the editorial line and are not obliged to report neutrally; they contain opinions, statements, and the evaluation of topics, actors, and current events [42]. Therefore, opinion pieces actively shape the social construction of reality and are also shaped by it [43, 44].

**Table 1:** Public communication of industry, science, and politics

Actor representatives		Website (last accessed on 31.12.2020)
Industry	Automobile manufacturer	
	VW	https://www.volkswagen-newsroom.com
	BMW	https://www.bmwgroup.com
	Suppliers	
	Bosch	https://www.bosch-presse.de/pressportal/de/de/news/
	Continental	https://www.continental.com/de
Science	Scopus	https://www.scopus.com/
	ScienceDirect	https://www.sciencedirect.com/
	FIDmove	https://www.fid-move.de/
Politics	Parties in German Bundestag	
	AfD	https://www.afdbundestag.de/
	CDU/CSU	https://www.cducsu.de/
	FDP	https://www.fdpbt.de/
	SPD	https://www.spdfraktion.de/
	Bündnis 90/Die Grünen	https://www.gruene-bundestag.de/
	Die Linke	https://www.linksfraktion.de/start/
	Committee on Transport and Digital Infrastructure in German Bundestag	
	Public hearings	https://www.bundestag.de/ausschuesse/a15_Verkehr/public_anhoerungen
	Printed matter	https://pdok.bundestag.de/
	Federal Ministry of Transport and Digital Infrastructure	https://www.bmvi.de/DE/Home/home.html

We defined the unit of analysis as the text material published between January 1st 2020, and December 31st 2020, containing the keyword "Verkehrswende" or "Mobilitätswende". This time frame was chosen because the mobility transition, its impacts and solutions were heavily discussed due to both the run-up to the federal elections and the Covid-19 pandemic. A total of 325 text documents were included in the analysis. Only for science as an actor, both English and German publications were collected and included in the study. This appeared to be reasonable to the extent that the scientific discourse in Germany is conducted to a large extent in English. Table 2 shows the distribution according to the respective actor.

**Table 2:** Frequency distribution of "Verkehrs/Mobilitätswende" in public communication between the actors

	Industry	Science	Politics	Media	Total
“Verkehrswende”	3	10	204	32	249
“Mobilitätswende”	14	5	45	12	76
Total	17	15	249	44	325

The operationalization of the four frame elements was central to the analysis. We remediated challenges resulting from vague and partly inconsistent definitions of the frame elements [45–47] by linking the elements with the levels and dynamics described in the MLP.

### Problem description

The frame element *problem definition* was included in our analysis as *problem description* since this term seemed more suitable for identifying those statements in the research material, where the mobility transition is framed as a challenge in the first place [45]. This element captures the support or opposition toward the Verkehrs/Mobilitätswende and their underlying reason for that. The problems described within the arguments were subdivided into (1) fundamental issues that either require transformative processes or (2) favour adherence to the status quo. Within the MLP, *problem descriptions* can be characterized as exogenous factors at the socio-technical landscape, e.g. climate change or the covid-19 pandemic or at the regime level, where problems can arise from prevailing and established structures and actions that also may generate influencing landscape-factors on the long term [31].

### Causal interpretation

The element *causal interpretation* was understood as a causal attribution of a problem in the past, present, or future [45]. Therefore, it was included as such in our analysis, addressing questions of whom or what the cause of a problem is ascribed. A cause can be attributed implicitly or explicitly. Implicitly, this frame element allows determining which individual or collective actors within the socio-technical regime are considered responsible for a problem. Explicitly, facts or circumstances are seen as accountable for a problem at the socio-technical landscape, or the regime level can be recorded. Thus, this frame element facilitates the understanding of either the socio-technical transition dynamics, or the concrete actors and/or issues responsible for such transitions.

### Problem intervention

Since other authors pointed out the ambiguous definition of *treatment recommendation*, we use *problem intervention* [45]. Within the research material, this element records the solutions or proposals for action to address measures concerning transitioning mobility*.* In addition, individual or collective actors who are held responsible for implementing this need for action can also be grouped under this frame element. The need for action must refer to a problem, might be directed at one or several actors simultaneously, and can vary in the intensity expressed. This allows us to understand the solutions and proposals stated in the research material related to implementing a mobility transition or maintaining the status quo. This also includes the actors held responsible at the regime or niche level and the emphasis given to the proposals.

### Moral evaluation

The frame element *moral evaluation* relates to a problem's causal factors and corresponding effects [28]. Accordingly, moral evaluations are made up of judgments of an individual or collective actor's past, present, or future actions and related value judgments concerning (assumed) intentions, properties, etc. [45]. A value judgment is understood as the intention of the actors' actions. Therefore, the element moral evaluation was included in our analysis in connection with a) *causal interpretation* and b) *problem intervention*. In both cases, individual or collective actors' (lack of) actions within a regime or a niche could be assessed based on their intentions.

These frame elements allowed for a structured evaluation of the data acquired from the websites and the newspapers by applying qualitative content analysis comprising a deductive–inductive formation of categories [48]. As a coding unit, semantic statements were defined, i.e. all statements addressing one of the four frame elements. First, the frame elements were established as deductive categories and passages directly referring to the frame elements were assigned accordingly. In a second step, these statements were subjected to comprehensive content analysis to inductively expand the categories through generalization, paraphrasing, and clustering, considering the thematic criteria of socio-technical transformations to reduce further the degree of abstraction [49]. The material was analysed with the software MAXQDA. The inter-coder reliability was tested as a goodness-of-fit criterion and was sufficient.

## Results

**Fig. 1:**
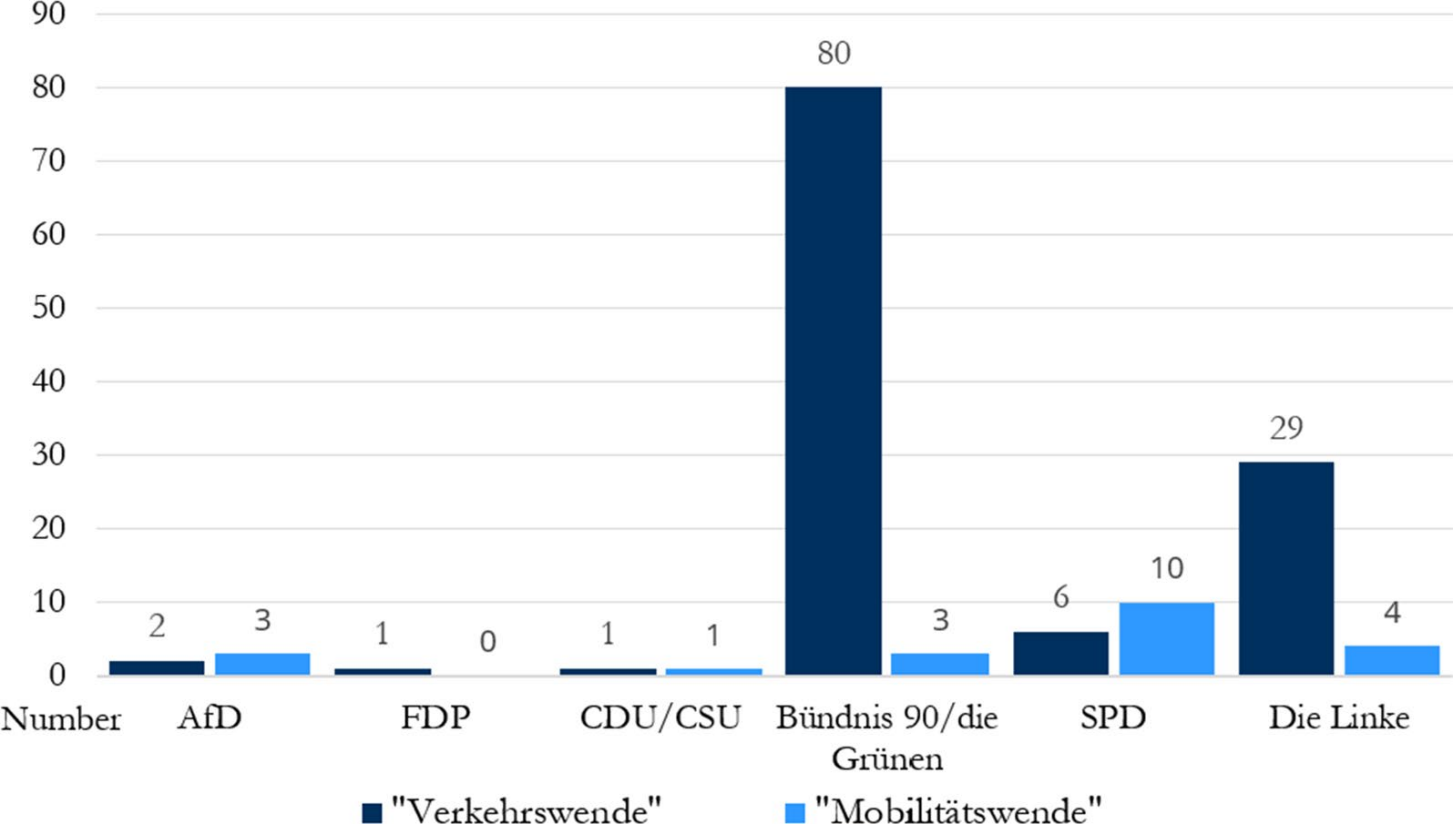
Frequency distribution of "Verkehrs/Mobilitätswende" on the websites of the political parties in the German Bundestag in 2020

For 2020, almost all selected representatives from industry, science, politics, and the media mentioned the term(s) "Verkehrs/Mobilitätswende" within their public communication. The only exception to this was found in the industry category with the automotive supplier *Continental*. A noticeable difference between parties was found within the political spectrum represented in the German Bundestag: Bündnis90/Die Grünen and Die Linke are the parties that most frequently include transitioning mobility in their agenda (see Fig. 1). Given the political interests of those parties, both left-wing, this is not surprising; conservative parties, on the other hand, rarely communicate about a transition of traffic and mobility.

### Problem description

Within the research material, actors define a concrete understanding of the term "Verkehrswende" or "Mobilitätswende" only occasionally; the terms are mainly used synonymously. Intermittently, the actors connect attributes such as *"sustainable"*, *"socio-ecological",* or *"affordable"* to the mobility transition. Additionally, the term *"transformation"* is sometimes used to refer to these attributes. To a great extent, the actors from industry, science, politics, and the media favour such a transformation in their communication, i.e. this is seen as necessary to achieve a particular state or result. In contrast, rejection—though possibly present in the more conservative actors—is rarely communicated and was thus recorded far less frequently.

#### Rejection of a Verkehrs/Mobilitätswende

In particular, conservative representatives from politics and the media made mention of reservations about current approaches to reach a Verkehrs/Mobilitätswende. Within the research material, neither science nor industry disapprove such transition. Rejections criticized previous implementation efforts, and attention is drawn to the missing consideration of emerging problems resulting from a mobility transition (Quote 1), the loss of individual freedom of motorists (e.g. driving bans) and economic consequences for the automotive industry (Quote 2). The politicization of climate targets and their inappropriateness or a lack of technology openness to alternative drives and fuels are mentioned, as well as a lack of profitability of such as well as further overall economic consequences. The main arguments here are the weakening of the German automotive industry and, as a result, the loss of jobs within this sector (Quote 3).

Quote 1: *"[...] the politically driven e-mobility transition is the main cause of the existential crisis in the German automotive industry [...]"* (FDP/11401)^2^

Quote 2: *"Once the bike takes over the road, it is difficult for the car to regain lost ground."* (Die Welt/32205)

Quote 3: *"What is astonishing is the heartlessness of some green actors towards workers, their families and the regions who have to pay the price for the urgently required change of mobility. Not all of them will find accommodation in bicycle workshops or as e-bus drivers."* (Die Welt/31201)

#### Approval of a Verkehrs/Mobilitätswende

The representatives listed various reasons why a mobility and transport transition is necessary. One of the main arguments concerned the environment and climate protection, voiced mainly (but not exclusively) by the parties Bündnis 90/Die Grünen and Die Linke: noise pollution, air pollution, climate change, the finiteness of fossil resources, CO_2_ emissions, and the achievement of national climate goals as well as the goals of the Paris Climate Agreement and the European Green Deal were explicitly mentioned (e.g. Quote 4). This issue, in particular, is hardly or not at all communicated by conservative actors such as the AfD party or the newspaper Die Welt. Infrastructural issues are also widely discussed. These primarily relate to the public transport regime, concerning the dismantling of railway lines, insufficient financial support for the expansion of public transport, and the lack of connection between peripheral and rural areas and cities. Another infrastructural issue was the lack of or poorly developed cycle paths. Here, the car itself or the concept of car-centrism is seen as the cause of the problem, as the infrastructure has mainly focused on the car, and other modes of transport have not been sufficiently taken into account in traffic planning (Quote 5). Together with the infrastructure, the study material also repeatedly refers to the lack of safety of current infrastructure in cities.

**Fig. 2:**
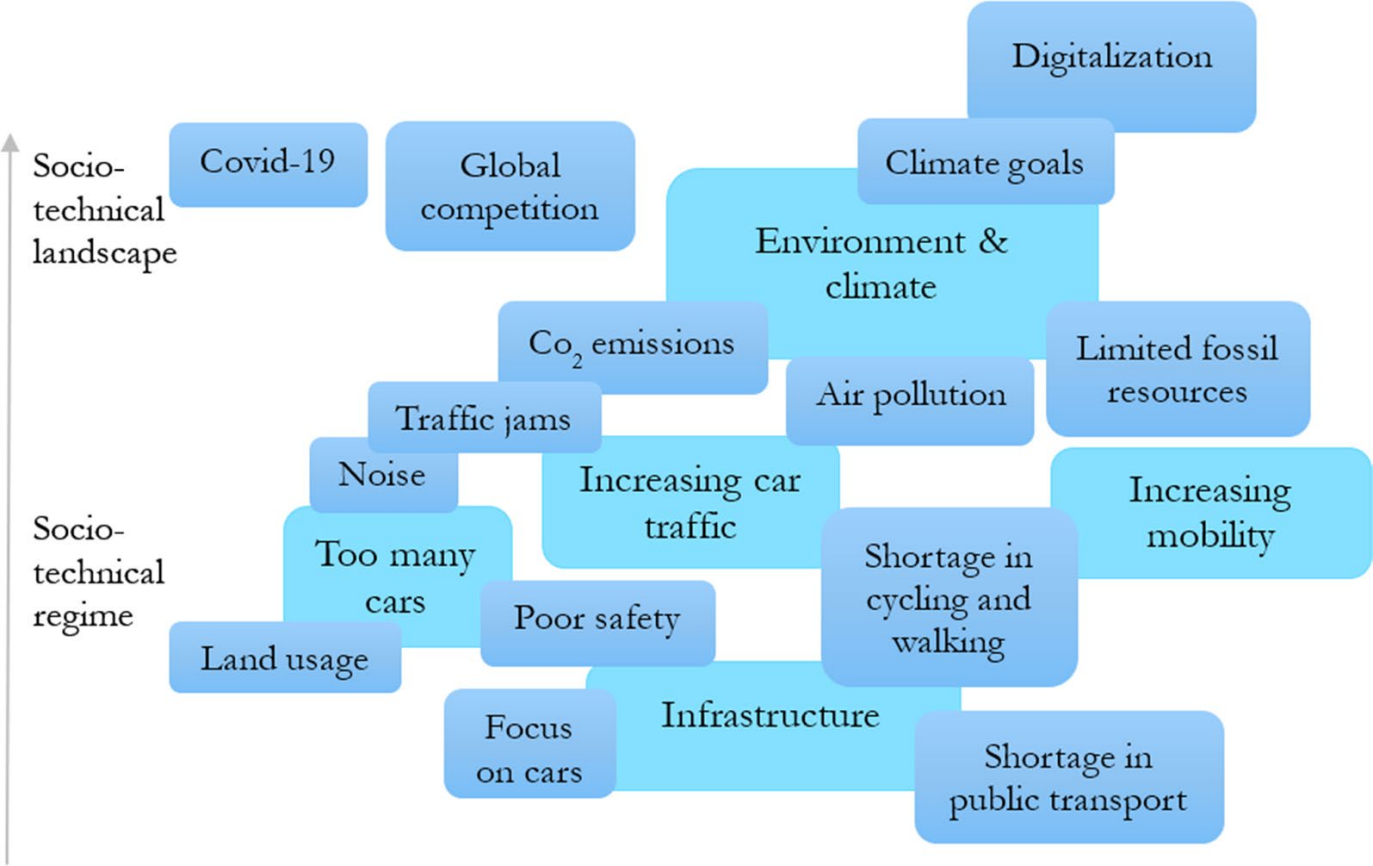
From the actors' point of view, problems within the transport and mobility system, that require a Verkehrs/Mobilitätswende. Own presentation based on Geels [29], lighter colour indicates main topics, darker colour related sub/topics

Regarding individuals' mobility, actors mention the increasing car traffic and the general increasing need for mobility. From an economic stance, actors reason with the safeguarding of jobs within the transport sector, the COVID-19 pandemic, and global competitiveness in the transport sector. Further, inadequate traffic safety and digitalization are mentioned (Quotes 6 and 7). See Fig. 2 for a summarized overview of the problem description.

Quote 4: *"We want cities that are bike-friendly, with good air to breathe, in which everyone is able to get around safely and in environmentally and climate-friendly ways. A mobility transition is overdue."* (Die Linke/12129)

Quote 5: *"But it is about a real turning point in transport policy: cyclists and pedestrians must finally get more space and therefore more protection in this city."* (Süddeutsche Zeitung/32305)

Quote 6: *"But we need a comprehensive Verkehrswende—not only to achieve the climate targets but also to deal with road congestion, noise pollution and air pollution."* (Volkswagen/42101)

Quote 7: *“The energy and mobility transition is central to the future of the location of business. Because this is where the topics of climate protection and future technologies, sustainability and competitiveness come together.”* (SPD/11206)

### Causal attribution and moral evaluation

Out of all frame elements in the material, *causal attribution* was found the least, with various actors and issues cited as causes. The following actors were identified: cyclists, motorists, the Deutsche Bahn, oil companies, political parties in the German Bundestag, the BMVI, and the minister of transport in 2020, Andreas Scheuer. The following issues were also identified: the historically evolved regime of automobility, too little or incorrectly made investments, and the prioritization of the private car, portrayed as out of date. However, the main causal attributions concerned the failed transport policy of the government and automobility in general. In most cases, these also received a *moral evaluation* by the actors.

#### Failed transport policy of the federal government

Various representatives see the federal government or its current and past transport policy and, in particular, the term of the minister of transport in 2020, Andreas Scheuer, as responsible for various problems that are currently perceived or communicated. This accusation is made mainly by the parties Bündnis 90/Die Grünen and Die Linke but was also found in the opinion pieces of the national daily newspapers Süddeutsche Zeitung and die tageszeitung. More specifically, the perceived backwardness of transport policy at the government level is criticized: automobile traffic is still the top priority in politics, the remaining infrastructure is neglected, and innovative and sustainable concepts are being pushed aside (Quotes 8 and 9). The representatives from science and industry criticize political processes rarely or not at all. Within science, only the relevance of politics for shaping transformation processes is pointed out. On the contrary, the automotive industry representatives included in this research have not commented on the prevailing transport policy.

Quote 8: *"That shouldn't hide the fact that the federal government is just getting ready to drive the Verkehrswende into the wall at full speed."* (taz/32403)

Quote 9: *"However, a very serious problem with regard to federal investments remains completely unsolved, namely that these investments still largely flow into old fossil fuel infrastructure."* (Die Linke/12112)

#### Criticism of automobility

Various actors view the hegemony of automobility as the cause of existing problems. Representatives of the media and the left-wing parties, in particular, state that too many cars in the city are the cause of bad air, noise, CO_2_ emissions, and problems with land use (Quote 10). Notably, *"the car"* or *"car traffic"* is often put forward as a cause; the behaviour of the car drivers is rarely discussed as an underlying concern (Quote 11). Of these, however, only society's technology handling is viewed critically; their technologies or environmentally harmful behaviour are not mentioned as a causal attribution for existing problems. In particular, national daily newspapers refer to people's change in mobility behaviour caused by the COVID-19 pandemic, highlighting existing issues and a Verkehrs/Mobilitätswende that appear necessary. The pandemic is seen as the cause of a behaviour change that has led to people a) driving more cars, b) cycling more, c) using public transport less, or d) generally driving less due to mobile work (Quote 12). The automotive industry is seldomly seen as the cause of existing problems; the role of the automotive industry is criticized almost exclusively by science. In the research material, the causes of current problems are mainly attributed to current or past transport policies and the hegemony of automobility. Advocates of the mobility transition mainly express criticism of the status quo.

Quote 10: *"We want a transport system that enables mobility in an environmentally friendly manner and works with less car traffic, especially in cities*." (SPD/11201)

Quote 11: *"The driver's heavy foot on the accelerator continues to have the greatest influence on energy consumption—as is the case today with gasoline and diesel vehicles."* (BMW/41203)

Quote 12: *"This is due to the standstill in Corona-land. In some cases, 90 per cent fewer passengers have recently boarded the trains. [...] Early surveys have already made clear that the importance of the car is increasing again in Germany in times of Corona. In order to avoid the risk of infection, more people will not only switch to bicycles but also to their own four wheels."* (SZ / 31303)

### Problem intervention and moral evaluation

**Fig. 3:**
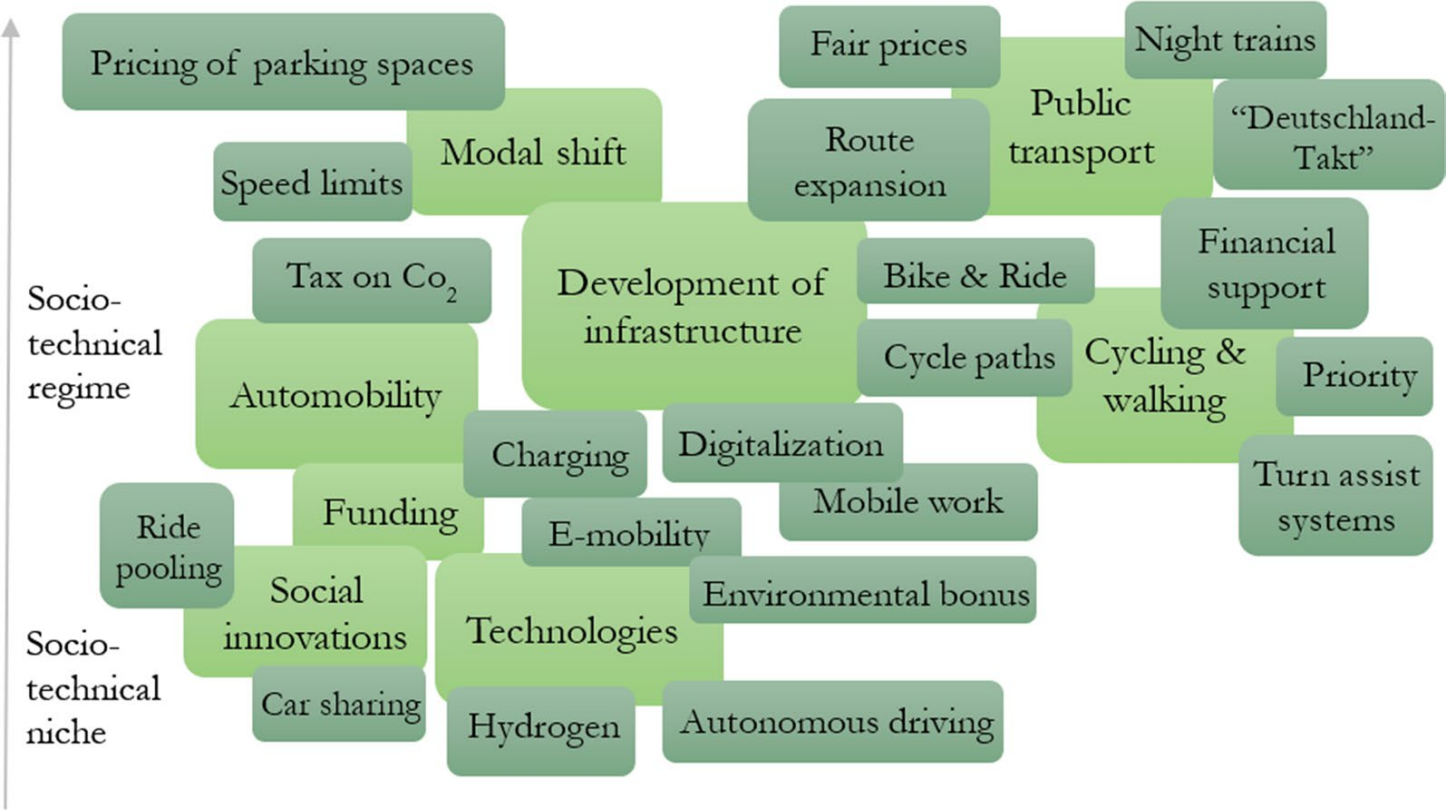
Actors communicated problem interventions across automobility to effect a Verkehrs/Mobilitätswende. Own representation based on Geels [29], lighter colour indicates main topics, darker colour related sub/topics

In the research material, most of the proposed solutions and actions were identified by the parties Bündnis 90/Die Grünen and Die Linke (also in the AVI). Occasionally, suggestions were made by the SPD, academia, and left-wing daily newspapers. Various actors suggested two main areas to solve the perceived problems: The improvement of propulsion technologies and a behavioural shift towards more sustainable modes of transport (which includes a structural shift away from automobility, too). For both, the expansion of various infrastructures was central. For a summarized overview of the problem intervention, see Fig. 3.

#### Improvement of propulsion technologies

Representatives of the automotive industry, and the governing parties in 2020, CDU/CSU and SPD, primarily point out the need for research on social and technical innovations to transform mobility and transport (Quote 13). The focus here is on maintaining automobility by promoting e-mobility and the corresponding charging infrastructure as well as hydrogen technologies and synthetic fuels. The latter is considered a solution by the FDP and the automotive industry (Quote 14). The topic of e-mobility within a transition in automobility is mainly viewed as critical by Bündnis 90/Die Grünen, e.g. concerning the sustainability of battery technology. Actors in the automotive industry or ruling politics in 2020 barely communicate about social innovations related to automobility, e-mobility, and hydrogen. Notably, within the MLP, improving propulsion technologies would only strengthen the established automobility regime and would not include social aspects that require changes in mobility behaviour or structures.

Quote 13: *"The sustainability and climate goals can only be achieved with research and innovations. Research not only creates basic knowledge and an orientation for setting goals. Research also brings about technological and social innovations and solutions, such as for the energy and transport transition, sustainable agriculture and forestry, sustainable urban development or sustainable economic activity and the future of work."* (AVI/CDU/CSU/12850)

Quote 14: *"The goal: The mobility of the future should not have any negative effects on the climate and air quality and should remain affordable for the general public. This is made possible with a mix of highly efficient combustion engines and state-of-the-art electric motors. In addition, Bosch advocates the use of regenerative and synthetic fuels so that the existing vehicle fleet can also contribute to reducing CO*_*2*_*."* (Bosch/41302)

#### Behavioural and structural shift

On the individual level, push and pull measures were proposed to support a behavioural change of mobility users. The parties Bündnis 90/Die Grünen and Die Linke mainly mentioned a behavioural shift away from the private car and towards other modes of transport, i.e. public transport, bicycle, and pedestrian traffic (Quote 15). The scientific community, left-wing politicians, and media representatives suggest push measures to achieve this behavioural shift, e.g. higher prices for public parking spaces, speed limits, or a CO_2_ tax. In terms of pull measures for public transport, the advocates mainly called for an expansion of the infrastructure, affordability, night trains, the reliable implementation of the "Deutschland-Takt", comfort in buses and trains, and a simplification of inter- and multimodality. Funding for public transport was also mentioned by the SPD (Quote 16). Apart from financial support, not many other concrete measures for implementation are communicated. For bicycle and pedestrian traffic, representative of the prevailing automotive industry, among others, surprisingly mentioned the expansion of cycle paths (Quote 17). In some cases, road safety improvement included proposals such as making trucks use turn assist systems compulsory or granting priority to cyclists and pedestrians in cities. The scientific community, left-wing parties, and media representatives also call for a shift away from the hegemony of automobility in road construction (Quotes 18 and 19). Notably, other representatives from the media and science hardly comment on this.

While these approaches can be categorized into push and pull measures, if implemented, they would simultaneously generate a structural shift away from the hegemony of automobility, too. Following the logic of the MLP, these solutions in the frame of behavioural changes would be the more sustainable solutions because they could lead to a more thorough change in the transport and mobility system as a whole. This claim is supported by the actors of science (Quote 20). Few statements link social and technical innovations within automobility, mentioned mainly by Bündnis 90/Die Grünen. This connection of social and technical innovations, however, according to the MLP, would be the most profound trajectory to achieve a shift within the transport and mobility system.

Quote 15: *"And it shows how the upcoming state aid and interventions could trigger a real change in mobility: Good and inexpensive connections by bus and train, with good conditions for walking and cycling, create many new jobs, improve the air and quality of life and contribute to environmental and climate protection.*" (Die Linke/11104)

Quote 16: *"That is why we are committed to promoting public transport. This includes investments in the railways, better networking of sharing offers, and promoting alternative fuels such as electromobility and hydrogen technology."* (SPD/11203)

Quote 17: *"In the corona crisis, Bosch E-Bike Systems sees politicians being called upon to lead the long-discussed mobility transition with determination and to expand the bicycle infrastructure in cities."* (Bosch/42301)

Quote 18: *"We demand: phasing out of the combustion engine by 2030. Reduction of climate-damaging subsidies for car, truck and air traffic. [...] Clear sanctions for driving too fast and a speed limit of 30 as the standard speed in city centres."* (Bündnis 90/Die Grünen/12678)

Quote 19: *"Instead of providing for new highways in this budget, instead of further promoting the combustion engine, instead of putting goods on the road, we need a priority in the area of environmental and climate protection."* (Die Linke/12106)

Quote 20: *"The central empirical thesis of the article is that, without politicisation of the transport issue and the involvement of labour, there will be no transition in transportation (in German: Verkehrswende) that clearly surpasses the narrow renewal of drivetrain technology offered by electric vehicles."* (Scopus/22102/p. 811)^3^

#### Responsibility attribution

The actors consider the Federal Government and the Ministry of Transport led by Andreas Scheuer in 2020 to be responsible for implementing specific measures or general requirements. Certain parties such as SPD, Die Linke, or Bündnis 90/Die Grünen consider themselves accountable for the implementation of the proposed measures (Quote 21). Mainly the latter refers to the role of society, municipalities, or urban planners, without whose assistance the proposed solutions cannot—in their opinion—be implemented. Social actors and the automotive industry are rarely seen as responsible for solving problems.

Quote 21: *"We need a second rail reform that will once again clearly define the role of the Deutsche Bahn in competition. This includes the federal government repositioning the DB Group in the medium term."* (Bündnis 90/Die Grünen/12660)

## Discussion

This paper aimed to understand how established actors from industry, science, politics, and the media publicly frame problems and solutions regarding the Verkehrs/Mobilitätswende in Germany. For this purpose, the frame elements of problem description, causal attribution, moral evaluation, and problem intervention were investigated. It is essential to consider that the results are highly influenced by the choice of representatives and by the time frame of investigation. Results indicate that all investigated actors discuss a transition of traffic and mobility. We found no analytical distinction between the concepts of "Verkehrswende" and "Mobilitätswende" within their public communication.

By analysing the frame element of *problem description*, it was possible, on the one hand, to determine that actors who address a mobility transition in their public communication support it more often than they reject it. The analysis also highlighted that actors use the frame element of *problem description* in a targeted manner following their economic or political interests: Either to emphasize relevant aspects or to avoid inapplicable arguments or the arguments of competing actors [27, 28, 47]. However, since the respective interests of the representatives were not included separately in the analysis, this can only be regarded as an indication. Regarding the approval and disapproval of a Verkehrswende, we found a transition was opposed only rarely. Only conservative opposition parties argue against a Verkehrs/Mobilitätswende, justifying this position with calls for protecting the freedom and independence of motorists. In this context, conservative political and media representatives argue that the Covid-19 pandemic would be used for transport policy purposes and to enforce a transition of mobility and transport. According to them, the ruling parties would abuse their positions of power, with motorists having to suffer from the consequences. Notably, no disapproval of a mobility transition was found in the research material for science or industry. As Heyen and Brohmann [50] point out, this is not surprising for science as an actor. However, this is much more surprising for industry as an actor, as it was represented by leading automotive sector companies in our study. Other than that, actors approve of a Verkehrswende, though the most insistent urge was found in the material of the "left-wing" parties.

Regarding the level of detail with which environmental and climate protection is dealt with, many differences are apparent: Above all, the parties Bündnis 90/Die Grünen and Die Linke address CO2 emissions and national and international climate protection goals. Within science, attention was primarily drawn to finite fossil resources and CO_2_ emissions as causes. In this regard, the results of our research go hand in hand with the perception within the scientific discourse that ecological aspects are seen as the most significant driver of transformation processes in transport and mobility [51]. The governing parties in 2020 CDU/CSU and SPD, on the other hand, also argue with environmental and climate protection for a mobility transition but do not go into any further details of this argument.

In sum, when actors from industry, science, politics, and the media communicate about Verkehrs/Mobilitätswende, they primarily support such a transformation. Most often, this advocacy is justified with environmental and climate protection. In this context, the dominant automobility regime and its impacts are seen as the leading root cause of the prevailing dilemmas in current transport policy. On the other hand, the hegemony of the car is held responsible for the inadequate infrastructure in other regimes such as public transport or bicycle and pedestrian traffic. Figure 2 shows an aggregated visualization of the above arguments mentioned by all actors. Linking trajectories, as well as the corresponding MLP levels, have been drawn and assigned.

Regarding causal attributions (which were identified less often than all other elements in the material), we conclude that the actors (1) either hardly question the problems mentioned, (2) do not consider the causes of the problems to be helpful for the respective argumentation, or (3) the causes are viewed by the actors as part of the problem itself and are therefore are not explicitly mentioned.

Notably, *"the car"* or *"car traffic"* is often put forward as a cause, but the behaviour of the car drivers is rarely discussed as an underlying concern. It is, however, striking that representatives of the automotive industry point this out.

Regarding the problem intervention, the proposed solutions and recommendations for action were manifold, indicating an understanding of the complexity of the topic. However, rather pessimistic actors about a transition of transport and mobility rarely formulated any problem solutions in the material. This seems plausible insofar as those actors in favour of a mobility transition strive to improve the status quo, while those who reject it tend to defend the status quo and view changes with concern [52]. In addition, the incumbent governing parties in 2020, i.e. SPD and CDU/CSU, suggest solutions, but these are not very differentiated or detailed. These representatives were previously responsible for transport policy in Germany and may therefore not want to discredit their work with detailed suggestions for improvement or may not see any far-reaching improvements in their work. The solutions and actions that were proposed concern (1) maintaining automobility or improving it, (2) shifting away from it, or (3) shifting to other regimes such as public transport or cycling and walking. Similar to Figs. 2, 3 shows an aggregated visualization of the abovementioned problem interventions drawn from the actors' discourse. Overlapping or adjacent squares correspond to linking trajectories stemming from diverse subtopics and leading towards main topics. Corresponding MLP levels have been assigned.

Accordingly, our study's results align with the literature on sustainable transport and mobility developments, where a distinction is made between improvement, shift, and reduction of trips and distances [53]. This, in turn, can be explained by the strong support of a mobility transition within the research material. On the other hand, the results indicate that actors in automobility are open to socio-technical innovations coming from niches such as car-sharing or e-mobility [54]. The focus on innovations such as e-mobility or hydrogen within the automobility regime suggests that these innovations are already being developed incrementally along established development paths [35].

Combining the MLP and the framing approach provided a fruitful path to better understanding actors' preferences, attitudes, and communication strategies in complex transformation processes such as the Verkehrs/Mobilitätswende. While the MLP provides a valuable framework for analysing the transition pathways and transformation trajectories caused by the mobility transition and its inherent challenges, adding the framing approach to the heuristic analysis allows one to better situate the various actors' positions and perceptions. Further, it enables one to understand whether the actors have a systemic understanding of transformation processes or if single and detached arguments that fit their interests are brought up in public communication. An example of a single argument would be promoting e-mobility as the solution for the mobility transition. Opposed to this, a systemic understanding would be conceptualizing e-mobility as part of a solution, but anticipating potential negative rebound effects caused by increased car use when comparatively inexpensive “fuel” is available and emissions appear negligible. Notably, the Verkehrs/Mobilitätswende as a term is even discussed in the regime of automobility. In politics, the term is mainly shaped by relatively progressive representatives as opposed to conservatives.

Our study only offers an analysis of the frames used around the notion of the mobility transition in Germany. While it provides an overview of possible transformation paths of transitioning mobility in Germany, all actors of the different regimes, not only selected representatives of automobility, would have to be included in future studies. Here, reference should be made to the definition deficit within the MLP, which initial empirical studies have already attempted to counteract [55]. Although the MLP offers a holistic view of socio-technical transitions, it is often argued that the dominant regime should consistently be considered together with other parallel existing regimes mentioned before (indicated in Fig. 3). Even though we just included actors to represent the automobility regime, these actors notably communicate beyond automobility and mention the regimes of public transport as well as pedestrians and bicycles. Therefore, we did not split up Fig. 3 according to mentioned solutions within single regimes. Here, our study is in line with the criticism of MLP at this point; the transport system needs to be considered as one system of transport and mobility. Thus, further research needs to determine which actors are representative of each regime and niche. In addition to the actor's selection, the sampling of the communication material was also limited by the global Covid-19 pandemic as the study considered the year 2020. Thus, the Covid-19 pandemic profoundly influenced transformative processes of traffic and mobility [30].

Another limitation of this study is the focus on mass media as the only indicator of public opinion. Mass media is crucial for the process of building public opinion and thus, it is significant for future (political) decisions and actions, but further analysis, e.g. surveys, must be carried out to incorporate primary data on opinions, knowledge, and behaviour of citizens across the different mobility regimes [41]. Moreover, future research could show the development of frames on the topic and the dynamics of actors within regimes and niches over time. Thus, a quantitative content analysis of mass media on this topic could not only account for the frames nuanced by different groups of citizens and society but also cater for the longitudinal effects.

## Conclusion

We conclude that the automobility regime represented in the study is only partially dynamic: various exogenous factors and consequences resulting from this prevailing regime are perceived as problems by the actors. By providing this overview of the different frame elements, our study highlights commonalities and differences within the actors' public communication. These problems are addressed and taken seriously as such; accordingly, they act as a driving force within transformation processes. The terms stand for somewhat different transformation processes from the actors' point of view and communication. Hence, the actors underline distinct niche developments and transformation trajectories and mention a wide variety of solutions to establish a level playing field for all mobility regimes and diminish the dominance of automobility.

Our study demonstrated that the actors do not have a common perception of problems. Therefore, they also do not use the terms Verkehrs/Mobilitätswende in a uniform way. Instead, the study highlighted that, above all, representatives of the actors industry, politics, and the media implicitly give the discourse a (political) direction by emphasizing relevant features and ignoring others according to their point of view, interests, and knowledge [27, 28, 47]. This is particularly reflected in the diversity of the problem descriptions and the corresponding problem interventions by the respective advocates.

The absence of a shared understanding of the underlying problems and solutions further aggravates the implementation gap of the mobility transition. In fact, the implementation gap is characteristic for many policy settings, in which incumbent actors are aiming at conquering the dominant regime of automobility and fostering transition processes towards sustainable mobility. This issue becomes even more relevant if implementation gaps are considered across the different governance levels. Our study focused on a sample of nationwide representatives active at the federal level, but frame elements used by actors in regimes and niches on other governance levels, such as the municipal or state level, could shed light on the mobility transition's implementation and communication gaps. Hence, this implementation gap goes hand in hand with the one in public communication, which this study tackles through a framing analysis of the notion of Verkehrs/Mobilitätswende. Although the mobility transition has been a central cornerstone of the coalition agreement of the new German Federal Government in 2021, implementing the necessary policy measures for this transition has been challenging. Analysing and understanding the frames used across the relevant regimes and niches is, therefore, key to reconciling the various problem definitions and reaching a consensus on this encompassing socio-technical transition.

## Data Availability

The datasets generated and/or analysed during the current study are available in "Framing "Verkehrs/Mobilitätswende" in Germany 2020: public communication of industry, science, media, and policy", Mendeley Data, V1" repository, https://doi.org/10.17632/ny3bgz5mmv.1.

## Appendix

Original quotations from the examined material in German language.

1. *„[…] die politisch forcierte E-Mobilitätswende, ist die Hauptursache der existenziellen Krise der deutschen Automobilindustrie […]“* (FDP/11401)
2. *„Wo einmal das Rad sich der Straße bemächtigt, gewinnt das Auto nur schwer verlorenes Terrain zurück.“* (Die Welt/32205)
3. *„Erstaunlich ist die Herzlosigkeit einiger grüner Akteure gegenüber Arbeitern, deren Familien und den Regionen, die den Preis für den so dringend angemahnten Mobilitätswechsel bezahlen müssen. Nicht alle von ihnen werden in Fahrradwerkstätten oder als E-Bus-Fahrer unterkommen.“* (Die Welt/31201)
4. *„Wir wollen fahrradgerechte Städte, mit guter Luft zum Atmen, in denen alle sicher, umwelt- und klimafreundlich unterwegs sind. Eine Verkehrswende ist überfällig.“* (Die Linke/12129)
5. *„Aber es geht doch um eine wirkliche Wende in der Verkehrspolitik: Fahrradfahrer und Fußgänger müssen in dieser Stadt endlich mehr Raum und damit auch mehr Schutz bekommen.“* (Süddeutsche Zeitung/32305)
6. *Wir brauchen aber eine umfassende Verkehrswende – nicht nur, um die Klimaziele zu erreichen, sondern auch um der Verstopfung der Straßen, der Lärmbelastung und der Luftverschmutzung beizukommen.“* (Volkswagen/42101)
7. *Zentral für die Zukunft des Wirtschaftsstandortes ist die Energie- und Mobilitätswende. Denn hier verbinden sich die Themen Klimaschutz und Zukunftstechnologien, Nachhaltigkeit und Wettbewerbsfähigkeit.* (SPD/11206)
8. *„Das darf nicht darüber hinwegtäuschen, dass sich die Bundesregierung gerade anschickt, die Verkehrswende mit Karacho vor die Wand zu fahren.“* (taz/32403)
9. *„Allerdings bleibt ein ganz gravierendes Problem, was die Investitionen des Bundes angeht, völlig ungelöst, nämlich dass diese Investitionen immer noch zum großen Teil in alte fossile Infrastruktur fließen.“* (Die Linke/12112)
10. *„Wir wollen ein Verkehrssystem, das Mobilität umweltgerecht ermöglicht und insbesondere in den Städten auch mit weniger Autoverkehr funktioniert*.“ (SPD, 11201)
11. *„Den größten Einfluss auf den Energieverbrauch hat nach wie vor der Gasfuß des Fahrers – sowie heute schon bei Benzinern und Diesel.“* (BMW/41203)
12. *„Schuld daran ist der Stillstand im Corona-Land. Zum Teil stiegen zuletzt 90 Prozent weniger Fahrgäste in die Züge. […] Erste Umfragen machen bereits deutlich, dass der Stellenwert des Autos in Corona-Zeiten in Deutschland wieder steigt. Um Ansteckungsrisiken aus dem Weg zu gehen, werden wieder mehr Menschen nicht nur auf das Fahrrad, sondern auch auf die eigenen vier Räder umsteigen.“* (SZ/31303)
13. *„Nur mit Forschung und Innovationen können die Nachhaltigkeits- und Klimaziele erreicht werden. Forschung schafft nicht nur Grundlagen- und Orientierungswissen zur Zielbestimmung. Forschung bringt auch technologische und Soziale Innovationen und Lösungen wie etwa für die Energie- und Verkehrswende, die nachhaltige Land- und Forstwirtschaft, die nachhaltige Stadtentwicklung oder ein nachhaltiges Wirtschaften und die Zukunft der Arbeit hervor.“* (AVI/CDU/CSU/12850)
14. *„Das Ziel: Die Mobilität der Zukunft soll keine negativen Auswirkungen auf Klima und Luftqualität haben und für die breite Bevölkerung bezahlbar bleiben. Möglich wird das mit einem Antriebsmix aus hocheffizienten Verbrennungs- und modernsten Elektromotoren. Zudem setzt sich Bosch für den Einsatz von regenerativen und synthetischen Kraftstoffen ein, damit auch der bereits vorhandene Fahrzeugbestand einen Beitrag zur CO2-Reduzierung leisten kann.“* (Bosch/41302)
15. *„Und es zeigt, wie die anstehenden staatlichen Hilfen und Eingriffe eine wirkliche Mobilitätswende anstoßen könnten: Gute und preiswerte Verbindungen per Bus und Bahn, mit guten Bedingungen für Fuß und Fahrrad schaffen viele neue Arbeitsplätze, verbessern Luft und Lebensqualität und tragen zu Umwelt- und Klimaschutz bei*.“ (Die Linke/11104)
16. *„Deshalb setzen wir auf die Förderung des öffentlichen Verkehrs. Das umfasst Investitionen in die Bahn, eine bessere Vernetzung von Sharing-Angeboten sowie die Förderung von alternativen Kraftstoffen wie Elektromobilität und Wasserstofftechnologie.“* (SPD/11203)
17. *„Bosch eBike Systems sieht die Politik in der Corona-Krise gefordert, die lang diskutierte Mobilitätswende mit Entschlossenheit voranzutreiben und die Fahrradinfrastruktur in den Städten auszubauen.“* (Bosch, 42301)
18. *„Wir fordern: Ausstieg aus dem fossilen Verbrennungsmotor bis 2030. Abbau klimaschädlicher Subventionen im Auto-, Lkw- und Flugverkehr. […] Klare Sanktionen für zu schnelles Fahren und Tempo 30 als Regelgeschwindigkeit in Innenstädten.“* (Bündnis 90/die Grünen/12678)
19. *„Statt neue Autobahnen in diesem Haushalt vorzusehen, statt den Verbrennungsmotor weiter zu fördern, statt Güter auf die Straße zu setzen, brauchen wir eine Priorität beim Bereich des Umwelt- und Klimaschutzes.* (Die Linke/12106)
20. Original quote from English publication, taken from Haas T (2020): Cracks in the gearbox of car hegemony: struggles over the German Verkehrswende between stability and change. Mobilities 15:810–827. https://doi.org/10.1080/17450101.2020.1817686.
21. *„Wir brauchen eine zweite Bahnreform, mit der die Rolle der Deutschen Bahn im Wettbewerb wieder klar definiert wird. Dazu gehört, dass der Bund den DB-Konzern mittelfristig neu aufstellt.“* (Bündnis 90/die Grünen/12660)

## Abbreviations

AfD: Alternative für Deutschland
AVI: Committee on Transport and Digital Infrastructure
BMW: Bayerische Motoren Werke Aktiengesellschaft
BMWI: Federal Ministry of Transport and Digital Infrastructure
CDU/CSU: Christlich Demokratische Union Deutschlands/Christlich-Soziale Union in Bayern e.V.
FAZ: Frankfurter Allgemeine Zeitung
FDP: Freie Demokratische Partei
FIDmove: Fachinformationsdienst Mobilitäts- und Verkehrsforschung
MLP: Multi-Level Perspective
SPD: Sozialdemokratische Partei Deutschlands
SZ: Süddeutsche Zeitung
taz: Die Tageszeitung
VW: Volkswagen

## Footnotes

1 German quotation, translated to English; translation has been verified by an official translator.

2 This and the following citations from the original German material were translated to English; translation has been verified by an official translator. The original quotes can be found in Appendix.

3 Original English publication, taken from Haas T (2020): Cracks in the gearbox of car hegemony: struggles over the German Verkehrswende between stability and change. Mobilities 15:810–827. https://doi.org/10.1080/17450101.2020.1817686.
